# Scent dog identification of SARS-CoV-2 infections in different body fluids

**DOI:** 10.1186/s12879-021-06411-1

**Published:** 2021-07-27

**Authors:** Paula Jendrny, Friederike Twele, Sebastian Meller, Claudia Schulz, Maren von Köckritz-Blickwede, Albertus Dominicus Marcellinus Eras Osterhaus, Hans Ebbers, Janek Ebbers, Veronika Pilchová, Isabell Pink, Tobias Welte, Michael Peter Manns, Anahita Fathi, Marylyn Martina Addo, Christiane Ernst, Wencke Schäfer, Michael Engels, Anja Petrov, Katharina Marquart, Ulrich Schotte, Esther Schalke, Holger Andreas Volk

**Affiliations:** 1 Department of Small Animal Medicine and Surgery, University of Veterinary Medicine Hannover, Bünteweg 9, 30559 Hannover, Germany; 2 Research Center for Emerging Infections and Zoonoses, University of Veterinary Medicine Hannover, Bünteweg 17, 30559 Hannover, Germany; 3 Department of Biochemistry, University of Veterinary Medicine Hannover, Bünteweg 17, 30559 Hannover, Germany; 4 KynoScience UG, Am Teutohang 51, 48477 Hörstel, Germany; 5 Department of Respiratory Medicine, Hannover Medical School, Carl-Neuberg-Straße 1, 30625 Hannover, Germany; 6 Hannover Medical School, Carl-Neuberg-Straße 1, 30625 Hannover, Germany; 7 Department of Medicine, Division of Infectious Diseases, University Medical-Center Hamburg-Eppendorf, Martinistrasse 52, 20246 Hamburg, Germany; 8 Department for Clinical Immunology of Infectious Diseases, Bernhard Nocht Institute for Tropical Medicine, Bernhard-Nocht-Straße 74, 20359 Hamburg, Germany; 9 German Center for Infection Research, Hamburg-Lübeck-Borstel-Riems, Germany; 10 Bundeswehr Medical Service Headquarters, Koblenz, Germany; 11 Bundeswehr School of Dog handling, Gräfin-Maltzan-Kaserne, Hochstraße, 56766 Ulmen, Germany; 12 Central Institute of the Bundeswehr Medical Service Kiel, Kronshagen, Germany

**Keywords:** SARS-CoV-2, Volatile organic compounds, Scent detection dogs, Saliva, Urine, Sweat

## Abstract

**Background:**

The main strategy to contain the current SARS-CoV-2 pandemic remains to implement a comprehensive testing, tracing and quarantining strategy until vaccination of the population is adequate. Scent dogs could support current testing strategies.

**Methods:**

Ten dogs were trained for 8 days to detect SARS-CoV-2 infections in beta-propiolactone inactivated saliva samples. The subsequent cognitive transfer performance for the recognition of non-inactivated samples were tested on three different body fluids (saliva, urine, and sweat) in a randomised, double-blind controlled study.

**Results:**

Dogs were tested on a total of 5242 randomised sample presentations. Dogs detected non-inactivated saliva samples with a diagnostic sensitivity of 84% (95% CI: 62.5–94.44%) and specificity of 95% (95% CI: 93.4–96%). In a subsequent experiment to compare the scent recognition between the three non-inactivated body fluids, diagnostic sensitivity and specificity were 95% (95% CI: 66.67–100%) and 98% (95% CI: 94.87–100%) for urine, 91% (95% CI: 71.43–100%) and 94% (95% CI: 90.91–97.78%) for sweat, 82% (95% CI: 64.29–95.24%), and 96% (95% CI: 94.95–98.9%) for saliva respectively.

**Conclusions:**

The scent cognitive transfer performance between inactivated and non-inactivated samples as well as between different sample materials indicates that global, specific SARS-CoV-2-associated volatile compounds are released across different body secretions, independently from the patient’s symptoms. All tested body fluids appear to be similarly suited for reliable detection of SARS-CoV-2 infected individuals.

**Supplementary Information:**

The online version contains supplementary material available at 10.1186/s12879-021-06411-1.

## Background

### Current situation

The recently emerged respiratory disease coronavirus disease 2019 (COVID-19) broke out in Wuhan, China, in December 2019 and was declared a global health emergency by the World Health Organization in January 2020 [1, 2]. The severe acute respiratory syndrome coronavirus 2 (SARS-CoV-2), which causes COVID-19, infects the upper respiratory tract and in more serious cases may also cause severe pneumonia and acute respiratory distress syndrome. The clinical presentation of SARS-CoV-2 infection is heterogeneous, ranging from asymptomatic infection to typical symptoms such as fever, cough, fatigue, ageusia and anosmia, but may also present atypically and lead to multiorgan dysfunction and death [1, 3, 4]. Containing this global pandemic requires a high rate of efficient testing, as an effective tool to contain viral spread. Viral loads can be detected by reverse transcription polymerase chain reaction (RT-PCR) assays and with slightly less sensitive and usually more rapid antigen detection tests in nasal or pharyngeal swabs [2, 4] and saliva [5–7] with a peak at days three to ten after infection. The peak of infectiousness is around symptom onset [8]. It remains unclear if sweat or urine are also sources of virus transmission [9, 10].

### Odour detection

Different infectious diseases may cause specific odours by emanating volatile organic compounds (VOCs). These are metabolic products, primarily produced by cell metabolism and released through breath, saliva, sweat, urine, faeces, skin emanations and blood [11]. The VOC-pattern reflects different metabolic states of an organism, so it could be used for medical diagnosis by odour detection and disease outbreak containment [12].

Canines are renowned for their extraordinary olfactory sense, being deployed as a reliable tool for real-time, mobile detection of, e.g., explosives, drugs and may identify certain pathogen- and disease-specific VOCs produced by infected body cells. The limit of detection for canines is at concentrations of one part per trillion, which is three orders of magnitude more sensitive than currently available instruments [12]. Consequently various studies have shown dogs‘abilities to detect with high rates of sensitivity and specificity [13] infectious and non-infectious diseases and conditions, such as different types of cancer [14], malaria [15], bacterial infections caused by e.g. *Clostridium difficile* or mastitis causing pathogens [16, 17], hypoglycaemia in diabetics [18], and virus infections in cell cultures [12, 19]. In addition, several research groups currently train and deploy SARS-CoV-2 detection dogs [20, 21]. In a pilot study, our group showed that dogs were able to detect inactivated saliva samples from COVID-19 patients with a sensitivity of 83% and specificity of 96% [22], which has been confirmed by other groups training dogs to detect either respiratory secretions or sweat samples from COVID-19 patients [20, 21]. Despite these preliminary promising results, it remains to be shown whether dogs detect VOCs which are biofluid-specific or alternatively there is a more general change in odour of COVID-19 patients. To test the latter hypothesis, the current study used the same training set-up with inactivated saliva samples as the former study [22]. The main objective of this study was to determine whether dogs trained with BPL (beta-propiolactone)-inactivated saliva samples of SARS-CoV-2 infected individuals are capable to detect native saliva samples of infected patients, as well as transfer recognition from saliva to other body fluids such as urine and sweat. The second aim was to investigate whether different stages of infection or clinical phenotypes of COVID-19 would alter the detection ability of the dogs.

Scent detection dogs could be a reasonable option for a first line screening method in public facilities or during major events as well as in medical institutions that would be real-time, effective, economical, effortless and non-invasive.

## Methods

### Samples - target scent, negative controls and distractors

To acquire saliva samples, individuals had to salivate about 1–3 ml through a straw into sample tubes. For the training phase, saliva samples from twelve subjects (hospitalised and non-hospitalised SARS-CoV-2 infected individuals) suffering from asymptomatic to severe COVID-19 symptoms were inactivated with beta-propiolactone (BPL) according to the protocol described in Jendrny et al. 2020 to provide safe training conditions for dogs and handlers. To generate sweat samples, the test persons had to wipe their crook of the arm with a cotton pad. Urine samples were collected from the test persons by urinating into a cup and transfer of 5 ml into a sample tube. After acquisition, all of the samples were deep-frozen at − 80 °C in the laboratory until usage. Samples from ninety-three participants (32 male and 61 female subjects) were used in the study (Additional Table 1). The SARS-CoV-2 status of each collected sample was determined by the RT-PCR SARS-CoV-2-IP4 assay from Institut Pasteur including an internal control system and protocol [23, 24].

In contrast to our first study [22], which only included hospitalised COVID-19 patients suffering from severe courses of disease, we now additionally included non-hospitalised asymptomatic individuals as well as individuals with mild clinical signs. Inclusion criteria were either the diagnosis of infection by positive SARS-CoV-2 RT-PCR of nasopharyngeal swabs (positive samples), negative SARS-CoV-2 RT-PCR result and healthy condition (negative control samples) or negative SARS-CoV-2 RT-PCR result and symptoms of other respiratory disease (distractor samples). Written consent from all participants were collected before sample collection. The local Ethics Committees of *Hannover Medical School* (MHH) and the Hamburg Medical Association (for the *University Medical-Center Hamburg-Eppendorf* (UKE)) approved the study (ethic consent number 9042_BO_K_2020 and PV7298, respectively).

To ensure safety for presentation of non-inactivated samples, glasses specially designed for scent dog training (Training Aid Delivery Device (*TADD*), Sci-K9, USA) containing an odour-permeable fluoropolymer membrane were used. A 1 × 1 × 0.5 cm cotton pad soaked with 100 μl of fluid sample material or a snippet of the cotton pad used for sweat sampling was placed at the bottom of the *TADD*-glass and the glass was safely sealed in the laboratory under biosafety level 3 laboratory conditions.

### Dogs

All ten dogs were German armed forces’ service dogs with a history of either protection work, explosives detection or no previous training except for obedience (Additional Table 2). Involved dog breeds were Malinois (*n* = 5), Labrador Retriever (*n* = 3), German Shepherd (*n* = 1) and a Dutch shepherd crossbreed dog (n = 1) with ages ranging between one and 9 years (median age = 3.7 years). Six female and four male dogs were included.

### Testing device

For the detection training and testing, a device called ‘Detection Dog Training System’ (DDTS, Kynoscience UG, Hörstel, Germany) was utilised, which provided automated and randomised sample presentations for the dogs as well as automatic rewards as described previously [22]. The recorded results were verified by manual video analysis.

### Training procedure

The training procedure was exclusively based on positive reinforcement. Dogs were familiarised to the DDTS for 6 days using a replacement odour, followed by specific training for 8 days to condition them for a SARS-CoV-2 specific odour in twelve inactivated positive saliva samples and negative control samples from healthy individuals, respectively. The final study was conducted on 4 days and included non-inactivated saliva samples as well as urine and sweat samples. All of the samples used in the final study had not been presented to the dogs before.

### Study design of the double-blinded study

The study was conducted in compliance with safety and hygiene regulations according to the recommendations of the Robert Koch Institute (Berlin, Germany), and approved by local authorities (regional health department and state inspectorate’s office; Hannover, Germany). All samples were handled by the same person with personal protective equipment to prevent odour contamination which may irritate the dogs. In the first session non-inactivated saliva samples were used to assess whether dogs were able to transfer their trained sniffing performance from inactivated to non-inactivated saliva samples. In the following sessions, the detection performances for non-inactivated sweat, urine, and, again, saliva samples were evaluated. There were four possibilities for the dogs to respond to the presented odours:

1. True positive (TP): the dog correctly indicates a SARS-CoV-2 positive sample
2. False positive (FP): the dog incorrectly indicates a negative control or distractor
3. True negative (TN): the dog sniffs shortly at a negative sample but correctly does not indicate it
4. False negative (FN): the dog sniffs shortly at a positive sample but does not indicate it

A detection trial was considered accomplished if the dog left his snout in the target scent presenting hole of the DDTS for ≥2 s, initiating automatically the reward-ejection of the device as well as the next randomised trial. By using the software-controlled DDTS, the dogs were automatically rewarded for indicating a positive sample without the study losing its double-blind status. In each trial, the device’s software randomly assigned the target scent’s position between the seven different positions without the dog or its handler knowing which hole was next positive. The results were recorded electronically for subsequent analysis and verified by manual time-stamped video analysis. The standard temperature in the dog training laboratory was controlled at 24 ± 1 °C.

Although the samples were presented to the dogs in safe specimen vessels (*TADD*-glasses), the detection experiments with infectious material were performed in a biosafety level 2 laboratory to prevent any risk of infection. After leaving the test room, the canines were washed with 4% chlorhexidine shampoo with at least ten min contact time to prevent any potential environmental contamination and virus spread. The equipment was disinfected after each test day with suitable disinfectant wipes soaked in limited virucidal disinfectant solution. In addition, swab samples of the dogs’ noses and from the outside of *TADD*-membranes were taken after each day of testing and examined with RT-PCR-assays at the Central Institute of the Bundeswehr Medical Service or Research Center for Emerging Infections and Zoonoses to exclude contamination and replication with infectious viral particles in the dogs’ noses or an escape of virus-containing material from the vessel (Additional Table 3).

### Analysis of sensitivity and specificity

Sample size and sample acquisition were conducted based on and according to our pilot study [22]. The diagnostic sensitivity as well as diagnostic specificity, positive predictive values (PPV), and negative predictive values (NPV) were calculated according to Trevethan [25]. 95% confidence intervals (CIs) for sensitivity, specificity, PPV, and NPV were calculated with the hybrid Wilson/Brown method [26]. Medians of sensitivity, specificity, PPV, NPV, and accuracy with corresponding 95% CIs of median were also calculated per session. Two-tailed Fisher’s exact test was used for analysis of the contingency tables; a *P* ≤ 0.05 was considered significant. All calculations were done with the Prism 9 software from GraphPad (La Jolla, CA, USA).

## Results

**Fig. 1:**
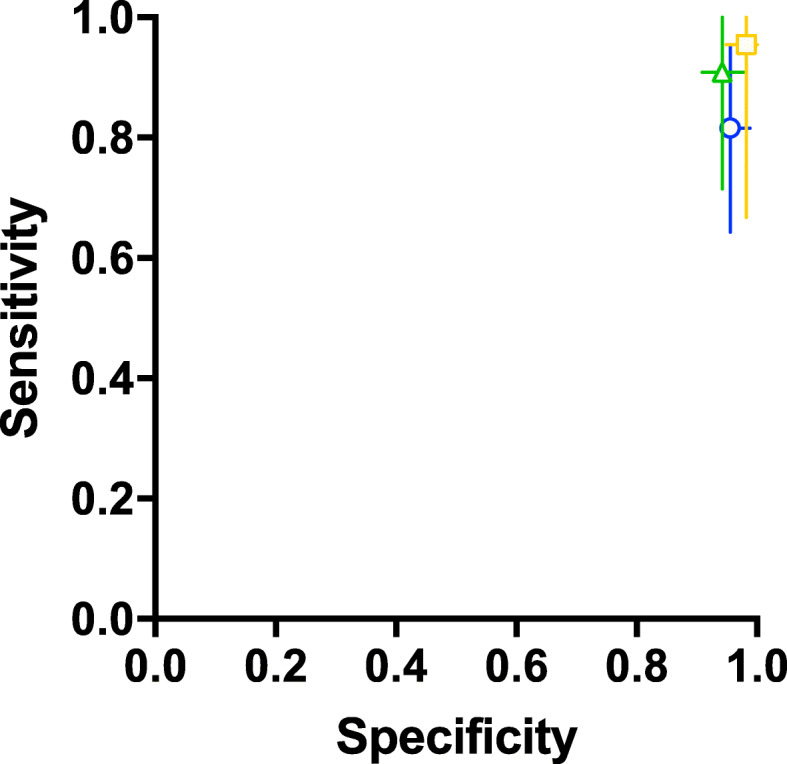
Median diagnostic specificity and sensitivity for all dogs for non-inactivated sweat (green triangle), urine (yellow square), and saliva (blue circle) samples, respectively. The 95% confidence intervals of the medians for specificity and sensitivity are shown with horizontal and vertical bars, respectively

When non-inactivated saliva samples were presented to the dogs after training with inactivated saliva samples, dogs were able to discriminate between samples of infected (RT-PCR positive), non-infected (RT-PCR negative) individuals and distractor samples (RT-PCR negative but respiratory symptoms) with a diagnostic sensitivity of 84% (95% CI: 62.5–94.44%) and specificity of 95% (95% CI: 93.4–96%). During the following detection sessions, when the device was equipped with non-inactivated samples with the same body fluid (saliva, sweat or urine), the corresponding values for diagnostic sensitivity and specificity for saliva samples were 82% (95% CI: 64.29–95.24%) and 96% (95% CI: 94.95–98.9%), for sweat samples 91% (95% CI: 71.43–100%) and 94% (95% CI: 90.91–97.78%), and for urine samples 95% (95% CI: 66.67–100%) and 98% (95% CI: 94.87–100%) respectively (Table 1, Fig. 1). Disease prevalence was about 18% on average.

**Table 1:** Diagnostic performance of the scent detection dogs

Session	Dog	Detection	SARS-CoV-2 infection status		Total number of sample presentations	Diagnostic specificity (Sp)	Diagnostic sensitivity (Se)	Confidence interval (95% CI) Sp	Confidence interval (95% CI) Se	Positive predictive value (PPV)	Negative predictive value (NPV)	Confidence interval (95% CI) PPV	Confidence interval (95% CI) NPV	Accuracy	Fisher’s exact test
			positive	negative											
**Non-inactivated saliva samples (after 1 week of training with inactivated saliva samples)**	Dog 1	Yes	15	5	132	0.9537	0.625	0.89618–0.98007	0.4271–0.78841	0.75	0.91964	0.5313–0.88814	0.8543–0.95715	0.89394	< 0.0001
		No	9	103											
	Dog 2	Yes	14	5	95	0.9375	0.93333	0.8619–0.97301	0.70183–0.99658	0.73684	0.98684	0.51208–0.88194	0.92916–0.99933	0.93684	< 0.0001
		No	1	75											
	Dog 3	Yes	16	5	136	0.95614	0.72727	0.90142–0.98112	0.51848–0.86849	0.7619	0.94783	0.54909–0.89372	0.89083–0.97587	0.91912	< 0.0001
		No	6	109											
	Dog 4	Yes	9	12	79	0.8209	0.75	0.71253–0.89446	0.46769–0.91106	0.42857	0.94828	0.2447–0.63453	0.85861–0.9859	0.81013	0.0002
		No	3	55											
	Dog 5	Yes	16	3	81	0.95161	0.84211	0.86712–0.98681	0.62435–0.9448	0.84211	0.95161	0.62435–0.9448	0.86712–0.98681	0.92593	< 0.0001
		No	3	59											
	Dog 6	Yes	16	5	107	0.94318	0.84211	0.8738–0.97549	0.62435–0.9448	0.7619	0.96512	0.54909–0.89372	0.90239–0.99049	0.92523	< 0.0001
		No	3	83											
	Dog 7	Yes	20	0	114	1	0.83333	0.95906–1	0.64147–0.93321	1	0.95745	0.83887–1	0.89564–0.98333	0.96491	< 0.0001
		No	4	90											
	Dog 8	Yes	17	3	92	0.96	1	0.88887–0.9891	0.81568–1	0.85	1	0.63958–0.94763	0.94935–1	0.96739	< 0.0001
		No	0	72											
	Dog 9	Yes	17	4	102	0.95238	0.94444	0.88387–0.98133	0.74243–0.99715	0.80952	0.98765	0.59999–0.92332	0.93333–0.99937	0.95098	< 0.0001
		No	1	80											
	Dog 10	Yes	14	7	129	0.93396	0.6087	0.86992–0.96765	0.40786–0.77842	0.66667	0.91667	0.45373–0.82805	0.84917–0.95554	0.87597	< 0.0001
		No	9	99											
						**Median Sp**	**Median Se**	**95% CI of median** **Sp**	**95% CI of median** **Se**	**Median PPV**	**Median NPV**	**95% CI of median** **PPV**	**95% CI of median NPV**	**Median** **accuracy**	**95% CI of median accuracy**
						**0.952**	**0.83772**	**0.93396–0.96**	**0.625–0.94444**	**0.7619**	**0.95453**	**0.66667–0.85**	**0.91964–0.98765**	**0.92558**	**0.87597–0.96491**
**Non-inactivated sweat samples**	Dog 1	Yes	10	0	67	1	0.55556	0.9273–1	0.33716–0.7544	1	0.85965	0.72247–1	0.74676–0.92713	0.8806	< 0.0001
		No	8	49											
	Dog 2	Yes	10	4	57	0.91489	1	0.80068–0.96641	0.72247–1	0.71429	1	0.45351–0.88279	0.91799–1	0.92982	< 0.0001
		No	0	43											
	Dog 3	Yes	10	5	69	0.90909	0.71429	0.80423–0.96054	0.45351–0.88279	0.66667	0.92593	0.41714–0.84824	0.82446–0.97082	0.86957	< 0.0001
		No	4	50											
	Dog 4	Yes	10	3	62	0.94231	1	0.84357–0.98428	0.72247–1	0.76923	1	0.49744–0.9182	0.9273–1	0.95161	< 0.0001
		No	0	49											
	Dog 5	Yes	10	7	71	0.87719	0.71429	0.76754–0.93922	0.45351–0.88279	0.58824	0.92593	0.36005–0.78389	0.82446–0.97082	0.84507	< 0.0001
		No	4	50											
	Dog 6	Yes	10	4	65	0.92157	0.71429	0.815–0.96908	0.45351–0.88279	0.71429	0.92157	0.45351–0.88279	0.815–0.96908	0.87692	< 0.0001
		No	4	47											
	Dog 7	Yes	10	1	44	0.9697	0.90909	0.84681–0.99845	0.62264–0.99534	0.90909	0.9697	0.62264–0.99534	0.84681–0.99845	0.95455	< 0.0001
		No	1	32											
	Dog 8	Yes	10	1	56	0.97778	0.90909	0.88433–0.99886	0.62264–0.99534	0.90909	0.97778	0.62264–0.99534	0.88433–0.99886	0.96429	< 0.0001
		No	1	44											
	Dog 9	Yes	10	1	40	0.96552	0.90909	0.82824–0.99823	0.62264–0.99534	0.90909	0.96552	0.62264–0.99534	0.82824–0.99823	0.95	< 0.0001
		No	1	28											
						**Median Sp**	**Median Se**	**95% CI of median** **Sp**	**95% CI of median** **Se**	**Median PPV**	**Median NPV**	**95% CI of median** **PPV**	**95% CI of median NPV**	**Median** **accuracy**	**95% CI of median accuracy**
						**0.94231**	**0.90909**	**0.90909–0.97778**	**0.71429–1**	**0.76923**	**0.96552**	**0.66667–0.90909**	**0.92157–1**	**0.92982**	**0.86957–0.95455**
**Non-inactivated urine samples**	Dog 1	Yes	10	1	91	0.98592	0.5	0.92444–0.99928	0.2993–0.7007	0.90909	0.875	0.62264–0.99534	0.78497–0.93066	0.87912	< 0.0001
		No	10	70											
	Dog 2	Yes	10	2	50	0.94872	0.90909	0.83114–0.99089	0.62264–0.99534	0.83333	0.97368	0.55197–0.97039	0.86505–0.99865	0.94	< 0.0001
		No	1	37											
	Dog 3	Yes	10	1	63	0.98	0.76923	0.89505–0.99897	0.49744–0.9182	0.90909	0.94231	0.62264–0.99534	0.84357–0.98428	0.93651	< 0.0001
		No	3	49											
	Dog 4	Yes	8	1	72	0.98333	0.66667	0.91145–0.99915	0.39062–0.86188	0.88889	0.93651	0.565–0.9943	0.84781–0.97503	0.93056	< 0.0001
		No	4	59											
	Dog 5	Yes	10	2	49	0.94872	1	0.83114–0.99089	0.72247–1	0.83333	1	0.55197–0.97039	0.90594–1	0.95918	< 0.0001
		No	0	37											
	Dog 6	Yes	10	2	66	0.96429	1	0.87881–0.99365	0.72247–1	0.83333	1	0.55197–0.97039	0.93359–1	0.9697	< 0.0001
		No	0	54											
	Dog 7	Yes	10	0	41	1	0.90909	0.88649–1	0.62264–0.99534	1	0.96774	0.72247–1	0.83806–0.99835	0.97561	< 0.0001
		No	1	30											
	Dog 8	Yes	10	0	47	1	1	0.90594–1	0.72247–1	1	1	0.72247–1	0.90594–1	1	< 0.0001
		No	0	37											
	Dog 9	Yes	10	0	49	1	1	0.91033–1	0.72247–1	1	1	0.72247–1	0.91033–1	1	< 0.0001
		No	0	39											
	Dog 10	Yes	10	4	66	0.92857	1	0.83025–0.97187	0.72247–1	0.71429	1	0.45351–0.88279	0.93121–1	0.93939	< 0.0001
		No	0	52											
						**Median Sp**	**Median Se**	**95% CI of median** **Sp**	**95% CI of median** **Se**	**Median PPV**	**Median NPV**	**95% CI of median** **PPV**	**95% CI of median NPV**	**Median** **accuracy**	**95% CI of median accuracy**
						**0.98167**	**0.95455**	**0.94872–1**	**0.66667–1**	**0.89899**	**0.98684**	**0.83333–1**	**0.93651–1**	**0.94959**	**0.93056–1**
**Non-inactivated saliva samples**	Dog 1	Yes	19	1	147	0.992	0.86364	0.95608–0.99959	0.66665–0.95251	0.95	0.97638	0.76387–0.99744	0.93285–0.99356	0.97279	< 0.0001
		No	3	124											
	Dog 2	Yes	20	5	128	0.95098	0.76923	0.89034–0.97888	0.57948–0.88966	0.8	0.94175	0.60869–0.91139	0.8787–0.97303	0.91406	< 0.0001
		No	6	97											
	Dog 3	Yes	20	7	173	0.95205	0.74074	0.90435–0.97658	0.55321–0.86830	0.74074	0.95205	0.55321–0.8683	0.90435–0.97658	0.91908	< 0.0001
		No	7	139											
	Dog 4	Yes	20	4	137	0.96522	0.90909	0.91397–0.98639	0.72185–0.98385	0.83333	0.9823	0.64147–0.93321	0.93776–0.99686	0.9562	< 0.0001
		No	2	111											
	Dog 5	Yes	20	5	120	0.94949	0.95238	0.88717–0.97824	0.77331–0.99756	0.8	0.98947	0.60869–0.91139	0.94276–0.99946	0.95	< 0.0001
		No	1	94											
	Dog 6	Yes	20	4	111	0.95506	0.90909	0.89007–0.98239	0.72185–0.98385	0.83333	0.97701	0.64147–0.93321	0.92001–0.99592	0.94595	< 0.0001
		No	2	85											
	Dog 7	Yes	18	8	163	0.94074	0.64286	0.88742–0.96967	0.45830–0.79294	0.69231	0.92701	0.50012–0.83499	0.87085–0.95987	0.88957	< 0.0001
		No	10	127											
	Dog 8	Yes	20	6	164	0.95556	0.68966	0.90643–0.97947	0.50770–0.82724	0.76923	0.93478	0.57948–0.88966	0.8807–0.96531	0.90854	< 0.0001
		No	9	129											
	Dog 9	Yes	20	1	112	0.98901	0.95238	0.94035–0.99944	0.77331–0.99756	0.95238	0.98901	0.77331–0.99756	0.94035–0.99944	0.98214	< 0.0001
		No	1	90											
	Dog 10	Yes	19	5	191	0.96875	0.6129	0.92894–0.98658	0.43824–0.76267	0.79167	0.92814	0.5953–0.90755	0.87861–0.95842	0.91099	< 0.0001
		No	12	155											
						**Median Sp**	**Median Se**	**95% CI of median** **Sp**	**95% CI of median** **Se**	**Median PPV**	**Median NPV**	**95% CI of median** **PPV**	**95% CI of median NPV**	**Median** **accuracy**	**95% CI of median accuracy**
						**0.95531**	**0.81644**	**0.94949–0.98901**	**0.64286–0.95238**	**0.8**	**0.96422**	**0.74074–0.9**	**0.92814–0.98901**	**0.93252**	**0.90854–0.97279**

During the presentation of 5308 randomised sample presentations, the overall success rate was 92% with 723 correct indications of positive, 4140 correct rejections of negative or distractors, 214 incorrect indications of negative and incorrect rejections of 231 positive sample presentations (Table 2).

**Table 2:** Detection performance and success rates per session and dog

Session	Dog	Detection	SARS-CoV-2 infection status		Total number of right decisions	Total number of sample presentations	Success rate per dog
			positive	negative			
**Non-inactivated saliva samples (after 1 week of training with inactivated saliva samples)**	Dog 1	Yes	15	5	118	132	89%
		No	9	103			
	Dog 2	Yes	14	5	89	95	94%
		No	1	75			
	Dog 3	Yes	16	5	125	136	92%
		No	6	109			
	Dog 4	Yes	9	12	64	79	81%
		No	3	55			
	Dog 5	Yes	16	3	75	81	93%
		No	3	59			
	Dog 6	Yes	16	5	99	107	93%
		No	3	83			
	Dog 7	Yes	20	0	110	114	96%
		No	4	90			
	Dog 8	Yes	17	3	89	92	97%
		No	0	72			
	Dog 9	Yes	17	4	97	102	95%
		No	1	80			
	Dog 10	Yes	14	7	113	129	88%
		No	9	99			
	All dogs	Yes	154	49	979	1067	92%
		No	39	825			
**Non-inactivated saliva, urine and sweat samples**	Dog 1	Yes	18	7	174	191	91%
		No	10	156			
	Dog 2	Yes	18	19	161	186	87%
		No	6	143			
	Dog 3	Yes	20	6	135	150	90%
		No	9	115			
	Dog 4	Yes	18	12	169	197	86%
		No	16	151			
	Dog 5	Yes	17	15	164	195	84%
		No	16	147			
	Dog 6	Yes	19	6	117	127	92%
		No	4	98			
	Dog 7	Yes	19	1	201	223	90%
		No	21	182			
	Dog 8	Yes	20	1	112	115	97%
		No	2	92			
	Dog 9	Yes	18	5	136	147	93%
		No	6	118			
	Dog 10	Yes	18	8	124	139	89%
		No	7	106			
	All dogs	Yes	185	80	1493	1670	89%
		No	97	1308			
**Non-inactivated sweat samples**	Dog 1	Yes	10	0	59	67	88%
		No	8	49			
	Dog 2	Yes	10	4	53	57	93%
		No	0	43			
	Dog 3	Yes	10	5	60	69	87%
		No	4	50			
	Dog 4	Yes	10	3	59	62	95%
		No	0	49			
	Dog 5	Yes	10	7	60	71	85%
		No	4	50			
	Dog 6	Yes	10	4	57	65	88%
		No	4	47			
	Dog 7	Yes	10	1	42	44	95%
		No	1	32			
	Dog 8	Yes	10	1	54	56	96%
		No	1	44			
	Dog 9	Yes	10	1	38	40	95%
		No	1	28			
	All dogs	Yes	90	26	482	531	91%
		No	23	392			
**Non-inactivated urine samples**	Dog 1	Yes	10	1	80	91	88%
		No	10	70			
	Dog 2	Yes	10	2	47	50	94%
		No	1	37			
	Dog 3	Yes	10	1	59	63	94%
		No	3	49			
	Dog 4	Yes	8	1	67	72	93%
		No	4	59			
	Dog 5	Yes	10	2	47	49	96%
		No	0	37			
	Dog 6	Yes	10	2	64	66	97%
		No	0	54			
	Dog 7	Yes	10	0	40	41	98%
		No	1	30			
	Dog 8	Yes	10	0	47	47	100%
		No	0	37			
	Dog 9	Yes	10	0	49	49	100%
		No	0	39			
	Dog 10	Yes	10	4	62	66	94%
		No	0	52			
	All dogs	Yes	98	13	562	594	95%
		No	19	464			
**Non-inactivated saliva samples**	Dog 1	Yes	19	1	143	147	97%
		No	3	124			
	Dog 2	Yes	20	5	117	128	91%
		No	6	97			
	Dog 3	Yes	20	7	159	173	92%
		No	7	139			
	Dog 4	Yes	20	4	131	137	96%
		No	2	111			
	Dog 5	Yes	20	5	114	120	95%
		No	1	94			
	Dog 6	Yes	20	4	105	111	95%
		No	2	85			
	Dog 7	Yes	18	8	145	163	89%
		No	10	127			
	Dog 8	Yes	20	6	149	164	91%
		No	9	129			
	Dog 9	Yes	20	1	110	112	98%
		No	1	90			
	Dog 10	Yes	19	5	174	191	91%
		No	12	155			
	All dogs	Yes	196	46	1347	1446	93%
		No	53	1151			
**All sessions**	Dog 1	Yes	72	14	574	628	91%
		No	40	502			
	Dog 2	Yes	72	35	467	516	91%
		No	14	395			
	Dog 3	Yes	76	24	538	591	91%
		No	29	462			
	Dog 4	Yes	65	32	490	547	90%
		No	25	425			
	Dog 5	Yes	73	32	460	516	89%
		No	24	387			
	Dog 6	Yes	75	21	442	476	93%
		No	13	367			
	Dog 7	Yes	77	10	538	585	92%
		No	37	461			
	Dog 8	Yes	77	11	451	474	95%
		No	12	374			
	Dog 9	Yes	75	11	430	450	96%
		No	9	355			
	Dog 10	Yes	61	24	473	525	90%
		No	28	412			
	All dogs	Yes	723	214	4863	5308	92%
		No	231	4140			

From 93 subjects in total, 46 were tested SARS-CoV-2 positive and 47 were tested SARS-CoV-2 negative via RT-PCR of nasopharyngeal swabs. The RT-PCR results of the sample material (saliva, sweat, urine) from participants with a diagnosed SARS-CoV-2 infection via nasopharyngeal swab and RT-PCR were only positive in twelve cases. The time interval between RT-PCR of the nasopharyngeal swab sample and RT-PCR of the sample material from the same individual ranged from 2 to 5 months, prior to which the sample material was stored and frozen at − 80 °C. Nasopharyngeal swabs from each dog, as well as from the outside of the membranes taken after each day of testing were all negative.

## Discussion

Rapid, affordable and accurate identification of SARS-CoV-2 infected individuals remains pivotal not only for limiting the spread of the current pandemic, but also for providing a tool to limit the impact on public health and the economy. Data from the current scent dog detection study confirm our former pilot study (sensitivity 84% versus 83% and specifity 95% versus 96%, respectively). In the current study, dogs were after only 8 days of training not only able to immediately transfer their scent detection abilities from inactivated to non-inactivated saliva samples, but also to sweat and urine, with urine having the highest sensitivity of 95% and specificity of 98%. These results suggest a general, non-cell specific, robust VOC-pattern generation in SARS-CoV-2 infected individuals and provide further evidence that detection dogs could provide a reliable screening method providing immediate results.

In the former pilot study from our group [22], only BPL-inactivated samples of COVID-19 patients and controls were used. The first step in the current trial was therefore to evaluate if dogs can transfer scent recognition to non-inactivated saliva samples, even when trained only with inactivated samples. The inactivation process with BPL did not impair the SARS-CoV-2-associated scent of the samples, as dogs were able to discriminate with a similar accuracy between inactivated and non-inactivated saliva samples from SARS-CoV-2 infected individuals and controls. This has implications for the training of dogs, as the health and safety measures other groups had to follow when using non-inactivated samples can be overcome by using BPL-inactivation. Data from the current study indicate that dogs can familiarise to a training device and be safely trained within little more than a week by using inactivated saliva samples from SARS-CoV-2 positive individuals and controls and become reliable SARS-CoV-2 detection dogs for untreated samples. Furthermore, the safety of working with the TADD-glasses was also confirmed by negative PCR results of the samples attained (canine nasopharynx and outer TADD-glass-membrane).

In a second step, untreated saliva, sweat and urine samples were presented to the dogs separately to evaluate if they can transfer scent recognition from saliva to other untreated body fluids. The detection rate for this experiment was also high, especially considering the dogs having not been trained with sweat or urine samples before. In order to eliminate the risk of recognizing an individual odour from a specific subject, samples used were different for each session.

The sample material of the individuals with positive SARS-CoV-2 status (nasopharyngeal swab tested positive via RT-PCR) was predominantly RT-PCR negative which could mean that dogs are able to detect the changes in metabolism of non-infectious secretions of SARS-CoV-2 infected individuals. This could explain some of the anecdotal reports from the scent detection work at Helsinki airport that dogs were able to detect asymptomatic SARS-CoV-2 infected individuals prior of them shedding virus. On the other hand, it is also possible that viral RNA has already degraded due to the storage process and is therefore no longer detectable via RT-PCR.

The fact that dogs were able to discriminate successfully between positive, negative samples and distractors represents evidence of a successful discrimination process, whereas the detection ability across three bodyfluids from 93 different individuals indicates a successful generalisation process. The study involves repeated measures since the same samples could be detected more than once in the same session. In any case, in the double-blind study, per detection cycle, dogs were confronted with samples they did not scent before and all negative and positive samples came from new and different patients.

Comparable to the current study, the prevalence in our pilot study was 18.5%. Furthermore, sensitivity and specificity were reproducible which was one of our goals. The high prevalence is due to the fact that always only one positive sample was presented next to several negative samples. It is important to note that prevalence is subject to dynamic processes and can impact predictive values of any screening method of a pandemic disease. Since the prevalence in our test paradigm is higher than in the current pandemic situation, with a growing number of people getting vaccinated, the real positive predictive values would be lower when sensitivity and specificity of dogs remain unchanged. In any case, a lower prevalence should not impact the performance of the dogs themselves, especially in the testing setting that we conducted, being rewarded with food for correct decisions. Level of frustration not finding a positive sample might increase when the prevalence falls below a certain threshold. Certainly, this ‘threshold of frustration’ depends on study design and mainly on personal traits of the dogs and training experience. However, the empty runs (presentation of only negative samples but no target scent) we used in training did not lead to excessive frustration in any of the dogs.

Several research groups that also trained SARS-CoV-2 sniffer dogs achieved good results, which support this work and consolidate the reliability of the canines’ olfactory sense for medical purposes. Grandjean et al. (2020) trained six dogs in 1 to 2 weeks using sweat samples and achieved success rates between 76 to 100% [20]. In addition to their work, where only sweat samples from hospitalised patients were used, the current study suggests that also asymptomatic SARS-CoV-2 infected individuals can be detected by the dogs. Our dogs were able to identify different COVID-19 disease phenotypes and phases of disease expression (sore throat, cough, cold, headache and aching limbs, fever, loss of smell and taste and/or severe pneumonia). Other scent dog detection studies, conducted by Vesga et al. (2020) and Eskandari et al. (2021) achieved promising results (Vesga et al.: 95.5% average sensitivity and 99.6% specificity; Eskandari et al.: 86% sensitivity and 93% specificity, respectively) and also planned real-life experiments [21, 27]. These studies support the evidence of canines offering a reliable screening method for SARS-CoV-2 infections. Future studies are important to address some remaining limitations such as the low number of distractor samples with specified pathogens (differentiation to other lung diseases or pathogens such as infections with other seasonal respiratory viruses, like influenza viruses, rhinoviruses, respiratory syncytial virus, human metapneumovirus, adenovirus, and coronaviruses other than SARS-CoV-2). This was however not within the scope of the current study. The laboratory identification of the specific VOC pattern is still in its infancy, but some current studies showed SARS-CoV-2 specific biomarkers in breath samples detectable by gas chromatography-ion mobility spectrometry [28, 29], which also support our hypothesis. Scent dogs should be considered an addition to the gold standard RT-PCR, for rapid testing in situations where great numbers of people from different origins come together. The accuracies may be increased by extending the training phase and selecting individual dogs with better scent detection accuracy. Dogs could also be trained to work directly on humans, but several factors need to be considered. People can be afraid of dogs, have strong allergies or be simply uncomfortable within the proximity of a dog. In addition, some infected individuals may feel stigmatized by being positively indicated by a dog. Therefore, the authors suggest a test scenario under real conditions as follows: Individuals to be tested should line up and swipe a cotton swab over the crook of their arm or neck. In the next step they present it to the dog through an opening in a partition wall which seperates the person to be tested from dogs as well as other individuals.

To date, there are very few reports of SARS-CoV-2 infections in dogs. Some studies confirm a limited susceptibility of the dog to this virus [30]. According to current data, SARS-CoV-2 could be detected in dogs via RT-PCR but seroconversion and mild clinical signs were also reported. However, serology in the canine population shows a very low prevalence [31]. Experimentally infected dogs neither shed nor spread the virus indicating no evidence regarding dog-to-human or dog-to-dog transmission [31]. Overall, these facts imply a low infection risk for working dogs.

As with any testing scenario, human and in this case dog daily performance could vary. This also applies to the most accurate diagnostic performance of the gold standard RT-PCR that can only be achieved under ideal conditions, which does not always reflects the real life situation. Peer reviewed and preliminary systematic reviews indicate PCR sensitivities ranging from 71 to 100% implying false negative results ranging up to 29% under real-life conditions [32, 33].

In order to generate rapid test results, a large number of over-the-counter rapid antigen tests are currently used. Test results are generated within about 15 min. According to the manufacturers, the tests approved in Germany have diagnostic sensitivities between 91 and 98% and specificities between 98 and 100% [34]. However, the diagnostic accuracy under real-life conditions is estimated to be much lower (pre-prints [35, 36]). A systematic review by Dinnes et al. (2021) evaluated the diagnostic accuracy of point-of-care antigen and molecular-based tests for SARS-CoV-2 infections and found sensitivities between 34.1 and 88.1% as well as an average specificity of about 99.6% [37] The Paul Ehrlich Institute (Langen, Germany) specified minimum criteria for approved rapid antigen test for SARS-CoV-2 infections. They require a diagnostic sensitivity of above 80% and specificity above 97% [38]. The scent dog method would meet these criteria. The purpose of validation of our screening method as a diagnostic test is out of the scope of the current study. The deployment of dogs as a real scenario SARS-CoV-2 screening method is just being implemented in some public facilities in different countries [39]. First reports are promising, however, further studies have to be implemented in order to validate dogs’ scent recognition capabilities as diagnostic tool for detection of SARS-CoV-2 infections.

## Conclusions

Detection dogs were able to transfer the conditioned scent of BPL-inactivated saliva samples to non-inactivated saliva, urine and sweat samples, with a sensitivity > 80% and specifity > 94%. All three fluids were equally suited for SARS-CoV-2 detection by dogs and could be used for disease specific VOC-pattern recognition. Detection dogs may provide a reliable screening method for SARS-CoV-2 infections in various settings to generate immediate results that can be verified by the gold standard (RT-PCR). Further work, especially under real-life conditions in settings where many individuals have to be screened is needed to fully evaluate the potential of the dog detection method.

## Supplementary Information

**Additional file 1: Additional table 1.** Characteristics of the samples used for the study.

**Additional file 2: Additional table 2.** Characteristics of dogs in the study.

**Additional file 3: Additional table 3.** Results of RT-PCR tests of TADD-membranes and dog noses after testing sessions.

## Data Availability

The datasets used and/or analysed during the current study are majorly included as additional information. Any additional data are available upon request from the corresponding author on reasonable request.

## Abbreviations

RT-PCR: Reverse transcription polymerase chain reaction test
VOCs: Volatile organic compounds
DDTS: Dog Detection Training System
BPL: Beta-propiolactone
TP: True positive
FP: False positive
TN: True negative
FN: False negative
CI: Confidence interval
TADD: Training Aid Delivery Device
