# Supplementary material for: Scent dog identification of SARS-CoV-2 infections in different body fluids
Source: BMC Infect Dis. 2021 Jul 27;21:707. doi: 10.1186/s12879-021-06411-1 (PMC8313882; doi:10.1186/s12879-021-06411-1)
Supplement: Supplementary file 1 — Additional file 1: Additional table 1. Characteristics of the samples used for the study. [file 12879_2021_6411_MOESM1_ESM.docx]

**Additional table 1.** Characteristics of the samples used for the study

| **Sample**  **number** | **Sample ID** | **Sex** | **Age (range in years)** | **Sample material** | **SARS-CoV-2 RT-PCR (swab)** | **SARS-CoV-2 RT-PCR (sample material)** | | | **Symptom status** | **Test type** |
| --- | --- | --- | --- | --- | --- | --- | --- | --- | --- | --- |
|  |  |  |  |  |  | SARS2-IP4-FAM | internal control EGFP 10^9-ML-HEX | result |  |  |
| 1 | PS2 | female | 20-30 | saliva | positive | N/A | N/A | N/A | severe* | Training (inactivated saliva) |
| 2 | NK-A | male | 40-50 | saliva | negative | N/A | N/A | N/A | none | Training (inactivated saliva) |
| 3 | PS7 | male | 60-70 | saliva | positive | N/A | N/A | N/A | severe* | Training (inactivated saliva) |
| 4 | NK-M | male | 50-60 | saliva | negative | N/A | N/A | N/A | none | Training (inactivated saliva) |
| 5 | PS8 | female | 40-50 | saliva | positive | N/A | N/A | N/A | severe* | Training (inactivated saliva) |
| 6 | NK-N | female | 40-50 | saliva | negative | N/A | N/A | N/A | none | Training (inactivated saliva) |
| 7 | PS6 | female | 50-60 | saliva | positive | N/A | N/A | N/A | severe* | Training (inactivated saliva) |
| 8 | NK-K | female | 50-60 | saliva | negative | N/A | N/A | N/A | none | Training (inactivated saliva) |
| 9 | PS9 | male | 60-70 | saliva | positive | N/A | N/A | N/A | severe* | Training (inactivated saliva) |
| 10 | NK-E | female | 30-40 | saliva | negative | N/A | N/A | N/A | none | Training (inactivated saliva) |
| 11 | PS10 | male | Okt 20 | saliva | positive | N/A | N/A | N/A | mild | Training (inactivated saliva) |
| 12 | NK-C | male | 30-40 | saliva | negative | N/A | N/A | N/A | none | Training (inactivated saliva) |
| 13 | PS11 | female | 40-50 | saliva | positive | N/A | N/A | N/A | mild | Training (inactivated saliva) |
| 14 | NK-H | female | 30-40 | saliva | negative | N/A | N/A | N/A | none | Training (inactivated saliva) |
| 15 | PS12 | male | 40-50 | saliva | positive | N/A | N/A | N/A | severe* | Training (inactivated saliva) |
| 16 | NK-I | female | 30-40 | saliva | negative | N/A | N/A | N/A | none | Training (inactivated saliva) |
| 17 | PS13 | male | Okt 20 | saliva | positive | N/A | N/A | N/A | severe* | Training (inactivated saliva) |
| 18 | NK-B | male | 30-40 | saliva | negative | N/A | N/A | N/A | none | Training (inactivated saliva) |
| 19 | PS14 | male | 70-80 | saliva | positive | N/A | N/A | N/A | severe* | Training (inactivated saliva) |
| 20 | NK-Q | male | 50-60 | saliva | negative | N/A | N/A | N/A | none | Training (inactivated saliva) |
| 21 | PS15 | male | 20-30 | saliva | positive | N/A | N/A | N/A | severe* | Training (inactivated saliva) |
| 22 | NK-F | female | 40-50 | saliva | negative | N/A | N/A | N/A | none | Training (inactivated saliva) |
| 23 | PS16 | male | 40-50 | saliva | positive | N/A | N/A | N/A | severe* | Training (inactivated saliva) |
| 24 | NK-G | female | 20-30 | saliva | negative | N/A | N/A | N/A | none | Training (inactivated saliva) |
| 25 | PS17 | male | Okt 20 | saliva | positive | 33.33 | 33.70 | positive | mild | Transfer inactive to active saliva |
| 26 | NK-T | female | 20-30 | saliva | negative | N/A | N/A | N/A | none | Transfer inactive to active saliva |
| 27 | PS71 | female | 20-30 | saliva | positive | 32.65 | 32.24 | positive | mild | Transfer inactive to active saliva |
| 28 | NK-U | female | 20-30 | saliva | negative | N/A | N/A | N/A | none | Transfer inactive to active saliva |
| 29 | T067 | female | 50-60 | saliva | negative | No Cq | 26.60 | negative | none | Transfer inactive to active saliva |
| 30 | PS25 | male | 40-50 | saliva | positive | 37.73 | 30.45 | positive | mild | Transfer inactive to active saliva |
| 31 | T079 | male | 40-50 | saliva | negative | No Cq | 26.88 | negative | none | Transfer inactive to active saliva |
| 32 | PS73 | male | 80-90 | saliva | positive | No Cq | 32.09 | negative | severe* | Transfer inactive to active saliva |
| 33 | T060 | male | 50-60 | saliva | negative | No Cq | 30.12 | negative | none | Transfer inactive to active saliva |
| 34 | PS30 | female | 20-30 | saliva | positive | 35.98 | 32.64 | positive | mild | Transfer inactive to active saliva |
| 35 | PS96 | female | 20-30 | saliva | negative | No Cq | 31.61 | negative | none | Transfer inactive to active saliva |
| 36 | PS61 | female | 50-60 | saliva | positive | No Cq | 33.33 | negative | asymptomatic | Transfer inactive to active saliva |
| 37 | PS95 | female | 20-30 | saliva | negative | No Cq | 30.48 | negative | none | Transfer inactive to active saliva |
| 38 | PS20 | female | 30-40 | saliva | positive | 26.60 | 31.40 | positive | mild | Transfer to urine and sweat |
| 39 | T031 | female | 30-40 | saliva | negative | No Cq | 26.53 | negative | none | Transfer to urine and sweat |
| 40 | PS21 | female | 40-50 | saliva | positive | No Cq | 31.49 | negative | mild | Transfer to urine and sweat |
| 41 | PS46 | female | 40-50 | saliva | negative | No Cq | 31.78 | negative | none | Transfer to urine and sweat |
| 42 | PS23 | male | 20-30 | urine | positive | No Cq | 29.85 | negative | mild | Transfer to urine and sweat |
| 43 | PS41 | male | 20-30 | urine | negative | No Cq | 29.63 | negative | none | Transfer to urine and sweat |
| 44 | PS63 | female | 20-30 | urine | positive | No Cq | 29.69 | negative | mild | Transfer to urine and sweat |
| 45 | PS52 | female | 20-30 | urine | negative | No Cq | 28.94 | negative | none | Transfer to urine and sweat |
| 46 | PS29 | female | 20-30 | sweat | positive | No Cq | 29.39 | negative | mild | Transfer to urine and sweat |
| 47 | PS42 | female | 20-30 | sweat | negative | No Cq | 29.44 | negative | none | Transfer to urine and sweat |
| 48 | PS31 | male | 30-40 | sweat | positive | No Cq | 29.70 | negative | mild | Transfer to urine and sweat |
| 49 | PS45 | female | 20-30 | sweat | negative | No Cq | 31.45 | negative | none | Transfer to urine and sweat |
| 50 | PS35 | female | 70-80 | sweat | positive | 37.35 | 29.17 | positive | asymptomatic | Transfer to urine and sweat |
| 51 | PS57 | female | 20-30 | sweat | negative | No Cq | 29.49 | negative | none | Transfer to urine and sweat |
| 52 | PS19 | male | Okt 20 | sweat | positive | No Cq | 31.54 | negative | asyptomatic | pure sweat |
| 53 | PS93 | female | 20-30 | sweat | negative | No Cq | 29.33 | negative | none | pure sweat |
| 54 | PS26 | female | 40-50 | sweat | positive | No Cq | 29.54 | negative | mild | pure sweat |
| 55 | PS53 | female | 20-30 | sweat | negative | No Cq | 31.75 | negative | none | pure sweat |
| 56 | PS77 | female | 70-80 | sweat | positive | No Cq | 31.36 | negative | severe* | pure sweat |
| 57 | PS82 | female | 30-40 | sweat | negative | No Cq | 31.65 | negative | none | pure sweat |
| 58 | PS70 | male | 50-60 | sweat | positive | No Cq | 30.07 | negative | severe* | pure sweat |
| 59 | PS84 | female | 30-40 | sweat | negative | No Cq | 31.23 | negative | none | pure sweat |
| 60 | PS68 | female | 50-60 | sweat | positive | 37.31 | 29.58 | positive | mild | pure sweat |
| 61 | PS81 | female | 20-30 | sweat | negative | No Cq | 29.58 | negative | none | pure sweat |
| 62 | PS39 | female | 50-60 | sweat | positive | No Cq | 29.94 | negative | mild | pure sweat |
| 63 | PS62 | female | 30-40 | sweat | negative (distractor) | No Cq | 29.90 | negative | mild | pure sweat |
| 64 | PS64 | male | 30-40 | sweat | positive | No Cq | 29.67 | negative | mild | pure sweat |
| 65 | PS85 | male | 20-30 | sweat | negative | No Cq | 32.04 | negative | none | pure sweat |
| 66 | PS65 | female | 30-40 | urine | positive | No Cq | 30.63 | negative | mild | pure urine |
| 67 | PS49 | female | 20-30 | urine | negative | No Cq | 34.06 | negative | none | pure urine |
| 68 | PS22 | male | Okt 20 | urine | positive | No Cq | 31.10 | negative | mild | pure urine |
| 69 | PS87 | male | 20-30 | urine | negative | No Cq | 29.68 | negative | none | pure urine |
| 70 | PS79 | female | 50-60 | urine | positive | No Cq | 31.08 | negative | mild | pure urine |
| 71 | PS86 | female | 20-30 | urine | negative | No Cq | 29.29 | negative | none | pure urine |
| 72 | PS36 | female | Okt 20 | urine | positive | No Cq | 29.39 | negative | mild | pure urine |
| 73 | PS94 | female | 20-30 | urine | negative | No Cq | 29.42 | negative | none | pure urine |
| 74 | PS27 | male | 40-50 | urine | positive | No Cq | 29.18 | negative | mild | pure urine |
| 75 | PS66 | male | 30-40 | urine | negative (distractor) | No Cq | 30.46 | negative | mild | pure urine |
| 76 | PS60 | male | 60-70 | urine | positive | No Cq | 33.30 | negative | severe | pure urine |
| 77 | PS88 | female | 20-30 | urine | negative | No Cq | 29.41 | negative | none | pure urine |
| 78 | PS97 | female | 20-30 | urine | positive | No Cq | 29.55 | negative | mild | pure urine |
| 79 | PS89 | female | 20-30 | urine | negative | No Cq | 31.42 | negative | none | pure urine |
| 80 | PS80 | female | 20-30 | saliva | positive | 28.40 | 29.47 | positive | mild | pure saliva |
| 81 | PS67 | female | 30-40 | saliva | negative (distractor) | No Cq | 30.55 | negative | mild | pure saliva |
| 82 | PS69 | female | 70-80 | saliva | positive | 30.68 | 32.74 | positive | severe* | pure saliva |
| 83 | T050 | female | 50-60 | saliva | negative | No Cq | 30.65 | negative | none | pure saliva |
| 84 | PS32 | male | 50-60 | saliva | positive | No Cq | 31.99 | negative | mild | pure saliva |
| 85 | T023 | male | 50-60 | saliva | negative | No Cq | 27.93 | negative | none | pure saliva |
| 86 | PS37 | female | Okt 20 | saliva | positive | 28.94 | 31.00 | positive | mild | pure saliva |
| 87 | T068 | female | Okt 20 | saliva | negative | No Cq | 27.12 | negative | none | pure saliva |
| 88 | PS76 | female | 70-80 | saliva | positive | No Cq | 32.44 | negative | severe* | pure saliva |
| 89 | T058 | female | 40-50 | saliva | negative | No Cq | 28.51 | negative | none | pure saliva |
| 90 | PS72 | female | 60-70 | saliva | positive | 21.27 | 30.48 | positive | severe* | pure saliva |
| 91 | PS18 | female | 30-40 | saliva | negative (distractor) | No Cq | 31.22 | negative | mild | pure saliva |
| 92 | PS78 | female | 70-80 | saliva | positive | 33.24 | 30.67 | positive | severe* | pure saliva |
| 93 | PS47 | female | 20-30 | saliva | negative | No Cq | 31.97 | negative | none | pure saliva |
|  |  |  |  |  |  |  |  |  |  |  |
| *hospitalised |  |  |  |  |  |  |  |  |  |  |
| N/A  not applicable |  | |  |  |  |  |  |  |  |  |
| Cq quantification cycle |  | |  |  |  |  |  |  |  |  |
