# Supplementary material for: Scent dog identification of SARS-CoV-2 infections in different body fluids
Source: BMC Infect Dis. 2021 Jul 27;21:707. doi: 10.1186/s12879-021-06411-1 (PMC8313882; doi:10.1186/s12879-021-06411-1)
Supplement: Supplementary file 2 — Additional file 2: Additional table 2. Characteristics of dogs in the study. [file 12879_2021_6411_MOESM2_ESM.docx]

**Additional table 2.**  Characteristics of dogs in the study

| **Name** | **Sex** | **Age (years)** | **Breed** | **Specialty** |
| --- | --- | --- | --- | --- |
| Lotta | female | 5 | Labrador Retriever | Explosives detection dog |
| Coyote | male, castrated | 9 | Dutch Shepherd Mix | Explosives detection and protection work |
| Donnie | male | 3 | Malinois | Explosives detection and protection work |
| Filou | female | 3 | Malinois | Mine detection dog |
| Füge | female, castrated | 4 | German Shepherd | no previous training except obedience |
| Vine | female, castrated | 5 | Malinois | no previous training except obedience |
| Bellatrix | female | 1 | Labrador Retriever | no previous training except obedience |
| Margo | female | 1 | Labrador Retriever | no previous training except obedience |
| Floki | male | 3 | Malinois | no previous training except obedience |
| Erec Junior | male | 3 | Malinois | Explosives detection and protection work |
