# Supplementary material for: Scent dog identification of SARS-CoV-2 infections in different body fluids
Source: BMC Infect Dis. 2021 Jul 27;21:707. doi: 10.1186/s12879-021-06411-1 (PMC8313882; doi:10.1186/s12879-021-06411-1)
Supplement: Supplementary file 3 — Additional file 3: Additional table 3. Results of RT-PCR tests of TADD-membranes and dog noses after testing sessions. [file 12879_2021_6411_MOESM3_ESM.docx]

**Additional table 3.** Results of RT-PCR tests of TADD-membranes and dog noses after testing sessions

| **Sample ID** | **Sample material** | **SARS-CoV-2 RT-PCR (swab)** | **SARS-CoV-2 RT-PCR (outside of the membrane)** | | | **Test type** |
| --- | --- | --- | --- | --- | --- | --- |
|  |  |  | SARS2-IP4-FAM | internal control EGFP 10^9-ML-HEX | result |  |
| NK-T | saliva | negative | No Cq | 31.63 | negative | Transfer inactive to active saliva |
| PS71 | saliva | positive | No Cq | 31.42 | negative | Transfer inactive to active saliva |
| NK-U | saliva | negative | No Cq | 33.89 | negative | Transfer inactive to active saliva |
| T067 | saliva | negative | No Cq | 31.55 | negative | Transfer inactive to active saliva |
| PS25 | saliva | positive | No Cq | 32.78 | negative | Transfer inactive to active saliva |
| T079 | saliva | negative | N/A | N/A | N/A | Transfer inactive to active saliva |
| PS73 | saliva | positive | No Cq | 31.99 | negative | Transfer inactive to active saliva |
| T060 | saliva | negative | N/A | N/A | N/A | Transfer inactive to active saliva |
| PS30 | saliva | positive | No Cq | 31.68 | negative | Transfer inactive to active saliva |
| PS96 | saliva | negative | N/A | N/A | N/A | Transfer inactive to active saliva |
| PS61 | saliva | positive | No Cq | 31.35 | negative | Transfer inactive to active saliva |
| PS95 | saliva | negative | N/A | N/A | N/A | Transfer inactive to active saliva |
| PS20 | saliva | positive | No Cq | 31.24 | negative | Transfer to urine and sweat |
| T031 | saliva | negative | No Cq | 31.13 | negative | Transfer to urine and sweat |
| PS21 | saliva | positive | No Cq | 31.85 | negative | Transfer to urine and sweat |
| PS46 | saliva | negative | No Cq | 31.74 | negative | Transfer to urine and sweat |
| PS23 | urine | positive | No Cq | 31.72 | negative | Transfer to urine and sweat |
| PS41 | urine | negative | No Cq | 31.53 | negative | Transfer to urine and sweat |
| PS63 | urine | positive | No Cq | 31.47 | negative | Transfer to urine and sweat |
| PS52 | urine | negative | N/A | N/A | N/A | Transfer to urine and sweat |
| PS29 | sweat | positive | No Cq | 31.29 | negative | Transfer to urine and sweat |
| PS42 | sweat | negative | N/A | N/A | N/A | Transfer to urine and sweat |
| PS31 | sweat | positive | No Cq | 31.38 | negative | Transfer to urine and sweat |
| PS45 | sweat | negative | N/A | N/A | N/A | Transfer to urine and sweat |
| PS35 | sweat | positive | No Cq | 31.98 | negative | Transfer to urine and sweat |
| PS57 | sweat | negative | N/A | N/A | N/A | Transfer to urine and sweat |
| PS19 | sweat | positive | No Cq | 31.61 | negative | pure sweat |
| PS93 | sweat | negative | No Cq | 31.85 | negative | pure sweat |
| PS26 | sweat | positive | No Cq | 31.87 | negative | pure sweat |
| PS53 | sweat | negative | No Cq | 31.89 | negative | pure sweat |
| PS77 | sweat | positive | No Cq | 32.15 | negative | pure sweat |
| PS82 | sweat | negative | No Cq | 31.82 | negative | pure sweat |
| PS70 | sweat | positive | No Cq | 31.51 | negative | pure sweat |
| PS84 | sweat | negative | N/A | N/A | N/A | pure sweat |
| PS68 | sweat | positive | No Cq | 31.24 | negative | pure sweat |
| PS81 | sweat | negative | N/A | N/A | N/A | pure sweat |
| PS39 | sweat | positive | No Cq | 31.10 | negative | pure sweat |
| PS62 | sweat | negative (distractor) | N/A | N/A | N/A | pure sweat |
| PS64 | sweat | positive | No Cq | 31.40 | negative | pure sweat |
| PS85 | sweat | negative | N/A | N/A | N/A | pure sweat |
| PS65 | urine | positive | No Cq | 33.20 | negative | pure urine |
| PS49 | urine | negative | No Cq | 31.79 | negative | pure urine |
| PS22 | urine | positive | No Cq | 31.86 | negative | pure urine |
| PS87 | urine | negative | No Cq | 32.44 | negative | pure urine |
| PS79 | urine | positive | No Cq | 31.37 | negative | pure urine |
| PS86 | urine | negative | No Cq | 31.79 | negative | pure urine |
| PS36 | urine | positive | No Cq | 31.52 | negative | pure urine |
| PS94 | urine | negative | No Cq | 32.18 | negative | pure urine |
| PS27 | urine | positive | No Cq | 31.26 | negative | pure urine |
| PS66 | urine | negative (distractor) | N/A | N/A | N/A | pure urine |
| PS60 | urine | positive | No Cq | 31.58 | negative | pure urine |
| PS88 | urine | negative | N/A | N/A | N/A | pure urine |
| PS97 | urine | positive | N/A | N/A | N/A | pure urine |
| PS89 | urine | negative | N/A | N/A | N/A | pure urine |
| PS80 | saliva | positive | No Cq | 32.40 | negative | pure saliva |
| PS67 | saliva | negative (distractor) | No Cq | 33.74 | negative | pure saliva |
| PS69 | saliva | positive | No Cq | 35.77 | negative | pure saliva |
| T050 | saliva | negative | No Cq | 34.00 | negative | pure saliva |
| PS32 | saliva | positive | N/A | N/A | N/A | pure saliva |
| T023 | saliva | negative | No Cq | 32.35 | negative | pure saliva |
| PS37 | saliva | positive | No Cq | 34.14 | negative | pure saliva |
| T068 | saliva | negative | N/A | N/A | N/A | pure saliva |
| PS76 | saliva | positive | No Cq | 31.97 | negative | pure saliva |
| T058 | saliva | negative | N/A | N/A | N/A | pure saliva |
| PS72 | saliva | positive | No Cq | 34.10 | negative | pure saliva |
| PS18 | saliva | negative (distractor) | No Cq | 35.32 | negative | pure saliva |
| PS78 | saliva | positive | No Cq | 31.80 | negative | pure saliva |
| PS47 | saliva | negative | N/A | N/A | N/A | pure saliva |

| **Dog** | **Testing day** | **SARS-CoV-2 RT-PCR (nasopharyngeal swab from dogs)** | | |
| --- | --- | --- | --- | --- |
|  |  | SARS2-IP4-FAM | internal control EGFP 10^9-ML-HEX | result |
| 1 | 1 | No Cq | 32.20 | negative |
| 2 | 1 | No Cq | 34.45 | negative |
| 3 | 1 | No Cq | 34.88 | negative |
| 4 | 1 | No Cq | 31.25 | negative |
| 5 | 1 | No Cq | 31.43 | negative |
| 6 | 1 | No Cq | 33.44 | negative |
| 7 | 1 | No Cq | 34.27 | negative |
| 8 | 1 | No Cq | 34.24 | negative |
| 9 | 1 | No Cq | 30.94 | negative |
| 10 | 1 | No Cq | 32.06 | negative |
| 1 | 2 | No Cq | 32.08 | negative |
| 2 | 2 | No Cq | 31.16 | negative |
| 3 | 2 | No Cq | 31.32 | negative |
| 4 | 2 | No Cq | 35.22 | negative |
| 5 | 2 | No Cq | 35.20 | negative |
| 6 | 2 | No Cq | 31.49 | negative |
| 7 | 2 | No Cq | 33.32 | negative |
| 8 | 2 | No Cq | 31.38 | negative |
| 9 | 2 | No Cq | 32.45 | negative |
| 10 | 2 | No Cq | 31.49 | negative |
| 1 | 3 | No Cq | 33.09 | negative |
| 2 | 3 | No Cq | 32.16 | negative |
| 3 | 3 | No Cq | 31.12 | negative |
| 4 | 3 | No Cq | 33.45 | negative |
| 5 | 3 | No Cq | 31.53 | negative |
| 6 | 3 | No Cq | 32.07 | negative |
| 7 | 3 | No Cq | 31.86 | negative |
| 8 | 3 | No Cq | 31.34 | negative |
| 9 | 3 | No Cq | 31.15 | negative |
| 10 | 3 | No Cq | 32.28 | negative |
| 1 | 4 | No Cq | 33.85 | negative |
| 2 | 4 | No Cq | 31.40 | negative |
| 3 | 4 | No Cq | 31.51 | negative |
| 4 | 4 | No Cq | 31.40 | negative |
| 5 | 4 | No Cq | 32.24 | negative |
| 6 | 4 | No Cq | 32.91 | negative |
| 7 | 4 | No Cq | 32.81 | negative |
| 8 | 4 | No Cq | 32.62 | negative |
| 9 | 4 | No Cq | 31.94 | negative |
| 10 | 4 | No Cq | 31.27 | negative |
| negative control | all days | No Cq | 32.09 | negative |
| positive control | all days | 29.29236301 | 40.45 | positive |
